# Content validation of the National Comprehensive Cancer Network/Functional Assessment of Cancer Therapy Lymphoma Symptom Index-18 (NFLymSI-18) in indolent B-cell non-Hodgkin’s lymphoma

**DOI:** 10.1186/s41687-024-00752-6

**Published:** 2024-07-09

**Authors:** Courtney N. Hurt, Karen Kaiser, Sara Shaunfield, Kimberly A. Webster, Karen Keating, Lara Boyken, Sara Duffey, Jessica Garcia, David Cella

**Affiliations:** 1 Department of Medical Social Sciences, Northwestern University Feinberg School of Medicine, Chicago, IL USA; 2 Bayer HealthCare Pharmaceuticals, Whippany, NJ USA; 3 Lurie Cancer Center, Northwestern University Feinberg School of Medicine, Chicago, IL USA

**Keywords:** FACIT, Lymphoma, Content validity, Concept elicitation, PRO development, Cognitive debriefing

## Abstract

**Background:**

The NFLymSI-18 is a patient-reported outcome measure comprised of the highest priority symptoms, emotional concerns, treatment side effects, and other concerns identified by lymphoma patients and oncologists. This study assessed the content validity of the NFLymSI-18 for patients with indolent B-cell non-Hodgkin’s lymphoma (iNHL), with a focus on the Disease-Related Symptoms Physical (DRS-P) subscale.

**Methods:**

Patients with a confirmed iNHL diagnosis who had received one or more lines of treatment were recruited during clinic visits. Patients described their symptoms, treatment side effects, and emotional concerns related to iNHL in a semi-structured interview. Qualitative data were analyzed using NVivo10.

**Results:**

Data saturation was obtained by the 18th interview. Most participants (67%) had follicular lymphoma. 28% of participants had marginal zone lymphoma, and one participant had lymphoplasmacytoid lymphoma/Waldenström macroglobulinemia. Mean age of the 18 participants was 67 years. 56% of the sample was male. Most participants (67%) had a college or advanced degree. When asked to describe their iNHL symptoms, patients most often discussed swelling (*n* = 14), fatigue (*n* = 11), and pain (*n* = 8). The following symptoms were mentioned by three patients each: anxiety, appetite loss, rash, sleep disruption, trouble breathing, and malaise. Mapping of NFLymSI-18 content to these concerns showed the instrument includes all those most frequently mentioned symptoms.

**Conclusions:**

This study supports the content validity of the NFLymSI-18, including its DRS-P Subscale, for patients with iNHL. The instrument shows strong validity for the most referenced symptoms of swelling, fatigue, and pain. The diversity of additional symptoms reported by patients is consistent with the heterogeneous symptomology of iNHL.

**Supplementary Information:**

The online version contains supplementary material available at 10.1186/s41687-024-00752-6.

## Background

Indolent non-Hodgkin’s lymphomas (iNHL) include a histologically diverse range of slow-growing lymphoproliferative malignancies characterized by a variety of clinical presentations [1, 2]. These types of lymphomas constitute approximately 40% of all lymphoma cases in the United States (U.S.) [3]. The disease can manifest with localized or systemic symptoms, depending on its extent and the specific affected sites or organs [4, 5]. Many patients are diagnosed without apparent symptoms and potentially have lived with the condition for years. Some patients may remain asymptomatic even with advanced disease [6–8]. Furthermore, due to the recurring nature of these cancers and periods of treatment-induced remission, patients can experience extended asymptomatic intervals [7, 9].

However, when iNHL symptoms are present, affected individuals may notice painless swelling (lymphadenopathy) of the lymph nodes in the neck, armpits, or groin. This swelling may be accompanied by lymphatic system involvement, affecting tissues in the thymus gland, spleen, tonsils, or bone marrow. Other symptoms include malaise, fatigue, reduced energy, and decreased appetite [1, 6, 10–13]. In some cases, large nodal lesions, often referred to as “bulky disease,” can induce compressive symptoms like pain, discomfort, or cosmetic concerns [10]. Severe night sweats, persistent fever, and unintentional weight loss comprise the three “B Symptoms” experienced by some patients with fast-growing lymphomas or with advanced disease [14]. Capturing iNHL symptoms through brief, clinically-relevant patient-reported outcome (PRO) measures enhances clinical research by providing a holistic understanding of treatment outcomes and patient experiences. This information is essential for improving patient-centered treatment decision-making, ensuring that treatments align with patient needs and ultimately advancing the field of iNHL therapy.

To address the need for brief and clinically relevant measures meeting criteria outlined in the U.S. Food and Drug Administration (FDA) Guidance for Industry on “*Patient-reported Outcome Measures: Use in Medical Product Development to Support Labeling Claims*,” [15] Cella and colleagues created a series of disease-specific symptom indexes for use in targeted oncology clinical trial endpoint assessments [16, 17]. Building on questionnaires in the Functional Assessment of Chronic Illness Therapy (FACIT) Measurement System [18–20] that had previously undergone extensive patient-centered development and validity testing, 11 tumor-specific symptom indexes (bladder, brain, breast, colorectal, head & neck, hepatobiliary, kidney, lung, lymphoma, ovarian, and prostate) were derived. The National Comprehensive Cancer Network—Functional Assessment of Cancer Therapy Lymphoma Symptom Index-18 (NFlymSI-18) [21] is one of the 11 indexes, for which the FACT-Lymphoma (FACT-Lym: FACT-G + Lymphoma specific subscale) served as the foundation. The NFlymSI-18 instrument contains 18 items divided into the following subscales: Disease Related Symptoms-Physical (DSR-P), Disease Related Symptoms-Emotional (DRS-E), Treatment Side Effects (TSE), and Functional Well-Being (FWB) [21].

Although the NFlymSI-18 was created with input from lymphoma patients and oncologists [21], and has been used in lymphoma clinical trials [22, 23], its validity in the context of B-cell iNHL has not yet been examined. To address the need for brief and focused clinically relevant measures for use in clinical research [15, 24], this study assessed the content validity of the NFLymSI-18, with emphasis on the 9-item DRS-P, for B-cell iNHL.

## Methods

### Recruitment of iNHL patients

**Fig. 1:**
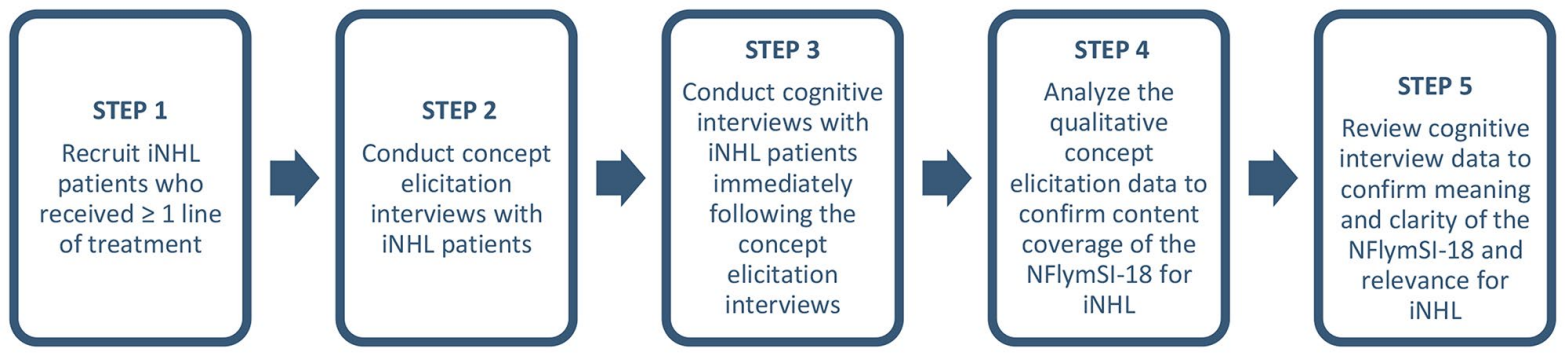
Methods Used to Evaluate Content Validity of the NFlymSI-18. *iNHL* indolent B-cell non-Hodgkin’s lymphoma, *NFlymSI-18* The National Comprehensive Cancer Network—Functional Assessment of Cancer Therapy Lymphoma Symptom Index-18

This study (STU00102531) was approved by the Northwestern University Institutional Review Board (IRB). A clinical recruitment specialist reviewed medical records at a large academic cancer center in the Midwest US to identify patients with a confirmed iNHL diagnosis who had received one or more lines of treatment. Eligible patients were approached in clinic prior to or following a scheduled appointment by a study team member, who explained the study using an IRB-approved recruitment script and obtained written informed consent from interested patients. The process for evaluating the content validity of the NFlymSI-18 is shown in Fig. 1 and described in greater detail below.

### Concept elicitation interview procedures

Interviews were held in the patient’s infusion room or in private meeting rooms. Interviews were conducted by trained, experienced interviewers (KK, LB, SS). Seven interviews were observed by a second interviewer for training purposes and quality assurance. All interviews were audio-recorded, with the participants’ permission, and recordings were transcribed.

The semi-structured interview guide was modeled after prior research [25–27]. First, basic sociodemographic and clinical information was collected. Then, patients were asked, “Please consider anything and everything that relates to your quality of life as you live with iNHL. What do you think is important in terms of your quality of life?” Patients were then asked to provide a detailed description of their symptoms. Interviewers took written notes of each symptom and asked clarifying questions, as needed. A series of probes were used to gather additional details about each symptom (e.g., “When did you first notice the symptom?”, “Can you describe that symptom in greater detail?”).

Patients were also asked to describe their treatment side effects and emotional concerns while interviewers took notes and asked probing questions to obtain detailed descriptions of each concern. During the interview, participants were asked to clarify when symptoms, treatment side effects, and emotional concerns developed and resolved. These details were compared with the patient’s diagnosis and treatment history, which were obtained from patient report and the medical record. Careful attention to timing enabled us to discern between treatment-related and disease-related concerns. Likewise, probes were used to obtain patient insights into the cause of the concern (e.g., disease symptom, treatment side effect, or other factors), which clarified concerns both proximal and distal to physical disease-related symptoms, which is the primary focus of this analysis.

### Cognitive interview procedures

After the concept elicitation interview, patients completed a cognitive interview. At the start of the cognitive interview, patients completed the NFlymSI-18 questionnaire while audio-recorded to capture any clarifying questions or comments. Using a cognitive interview guide, based on Willis [28], comprehension of the NFlymSI-18 was assessed in two parts: Part 1 examined recall period, instruction clarity, response option appropriateness, and questionnaire length. Part 2 involved item-by-item debriefing. Patients were asked to explain their answer to each item and restate the item in their own words. They were also asked if the meaning of the item was clear, if they had any questions or concerns, and if the item was relevant to their iNHL experience. Detailed field notes were maintained and entered into an Excel spreadsheet post-interview.

### Sample size

The estimated sample size required to achieve theoretical saturation [29], or the point in which no new relevant information is reported for three consecutive concept elicitation interviews, is between 12 and 24 participants [30, 31]. Based upon prior experience conducting cognitive interviews of newly drafted PRO measures, we anticipated that 10–12 cognitive interviews would be sufficient.

### Analysis

Concept elicitation interview data were analyzed using a constant comparative approach [32]. First, we reviewed detailed notes from the Excel file and compiled a preliminary list of quality-of-life concerns, symptoms, side effects, and emotional issues, removing redundant concepts. The resulting concepts were structured into a preliminary codebook. Next, three data analysts independently reviewed and coded three transcripts in NVivo10 using the draft codebook, noting missing or problematic codes. In group meetings, the coded transcripts were collectively reviewed, discrepancies were discussed, and the codebook was refined.

After finalizing the codebook, two team members independently coded the remaining transcripts. After all transcripts were coded, data for each code was extracted and reviewed in a coding review process whereby two team members individually reviewed each code. Data summaries for each code were composed, and any coding discrepancies were resolved through team discussion and corrected.

The final qualitative results were mapped to the NFlymSI-18. While patients were asked about concepts outside of the DRS-P, the primary interest for this analysis was the content validity of the DRS-P subscale. This process iteratively compared interview data with instrument content to identify (1) key themes from qualitative interviews covered by the NFlymSI-18 DRS-P; (2) NFlymSI-18 DRS-P content misaligned with qualitative data; and (3) important themes not reflected in the NFlymSI-18 DRS-P. A strong content validity match need not be inclusive of all patient-provided concepts, but should represent the vast majority of patient input [33].

For the cognitive interview data analysis, the study team met weekly to review the data for each item of the questionnaire and to discuss preliminary findings. After all data were entered into Excel, a final item-level data summary was created with the following information: (1) number of patients who interpreted the item in an unanticipated way (with description of those interpretations); (2) number of patients who said the meaning of the item was clear/unclear; (3) number of patients who said the item was/was not relevant to them. Additional information, such as patient feedback on the meaning of a specific word or phrase was also reviewed and summarized.

## Results

**Fig. 2:**
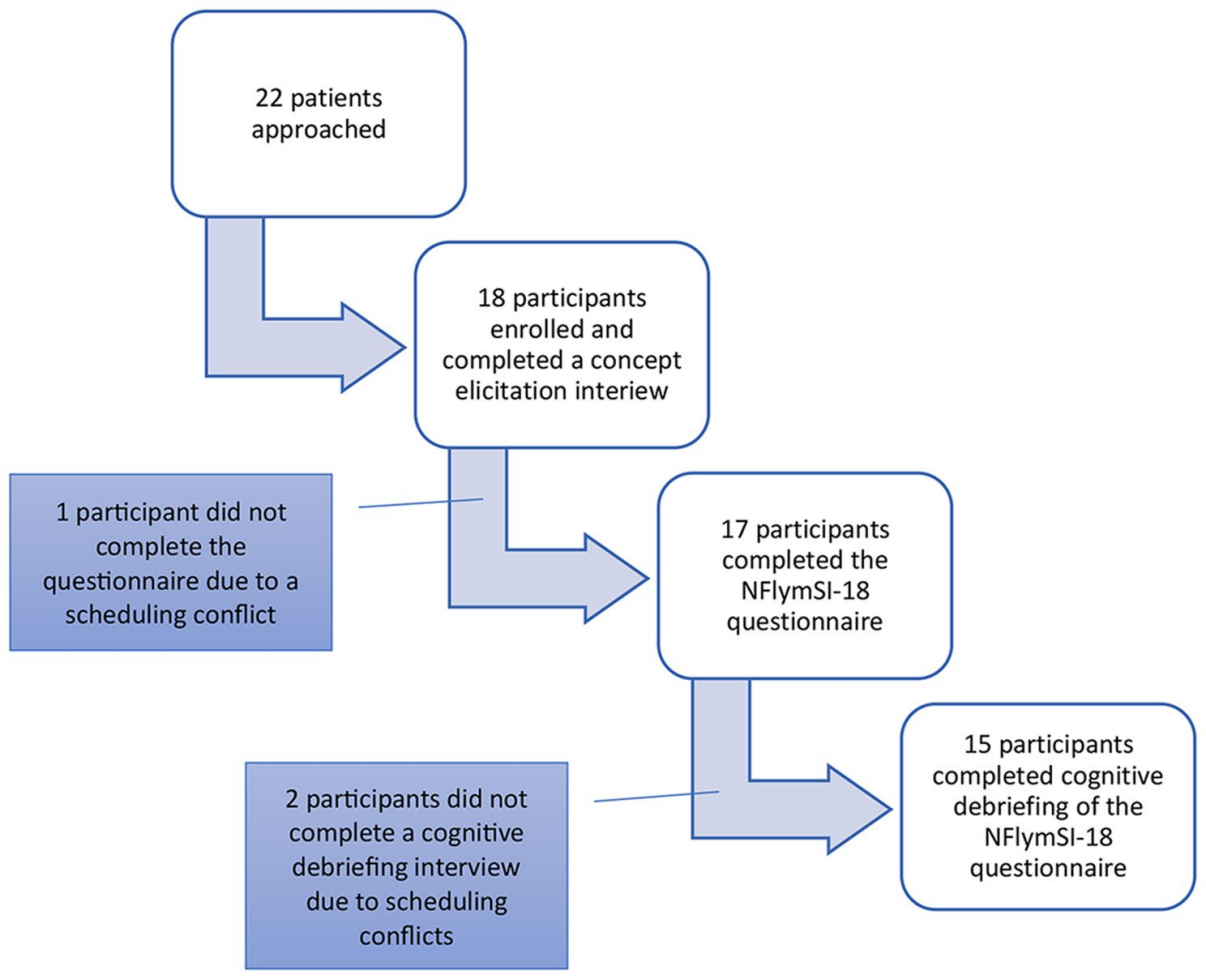
Study participant flow diagram

Twenty-two patients were approached and invited to enroll in the study. Of these, 18 participants completed a concept elicitation interview. Seventeen participants completed the FLymSI-18 questionnaire, and of these, 15 completed questionnaire cognitive debriefing. Figure 2 is a flow diagram of study participants.

Mean age of the 18 patients who completed concept elicitation interviews was 66.9 years. 56% of the sample was male. 83% of the sample was White. Most participants (66.7%) had a college-level or advanced degree. 89% of the sample had a self-reported Eastern Cooperative Oncology Group (ECOG) Performance Status ≤ 1 [34]. Interviews lasted 45 min, on average. Participant characteristics are shown in Table 1.

**Table 1:** Study participant characteristics

Characteristic	Concept elicitation (*n* = 18)	Cognitive debriefing (*n* = 15)
	Mean (range, median)	Mean (range, median)
Age	66.8 (49–85, 67)	66.3 (49–81, 66)
NFlymSI-18 score^a^		
Total score	58.5 (37–70, 61)	57.5 (37–70, 59)
DRS-P subscore	29.9 (20–36, 31)	29.1 (20–36, 31)
DRS-E subscore	12 (3–16, 12)	11.8 (3–16, 12)
TSE subscore	10.5 (8–12, 11)	10.7 (8–12, 11)
F/WB subscore	6.1 (0–8, 7)	5.9 (0–8, 7)
Gender		
Male	10 (55.6%)	7 (46.7%)
Female	8 (44.4%)	8 (53.3%)
Education		
Some high school	1 (5.6%)	1 (6.7%)
High school	3 (16.7%)	3 (20.0%)
Some college	2 (11.1%)	2 (13.3%)
College	5 (27.8%)	5 (33.3%)
Advanced degree	7 (38.9%)	4 (26.7%)
Ethnicity		
Hispanic or Latino origin	2 (11.1%)	2 (13.3%)
Non-Hispanic or Latino origin	16 (88.9%)	13 (86.7%)
Race		
White	15 (83.3%)	12 (80.0%)
African-American or Black	2 (11.1%)	2 (13.3%)
Other (biracial)	1 (5.6%)	1 (6.7%)
ECOG Status (Self-reported)^b^		
0	11 (61.1%)	9 (60.0%)
1	5 (27.8%)	4 (26.7%)
2	2 (11.1%)	2 (13.3%)
Disease histology		
Follicular Lymphoma	12 (66.7%)	10 (66.7%)
Marginal zone lymphoma	5 (27.8%)	4 (26.7%)
Lymphoplasmacytoid lymphoma/Waldenström macroglobulinemia	1 (5.6%)	1 (6.7%)
Treatment at time of interview		
Receiving treatment^c^	11 (61.1%)	9 (60.0%)
No treatment	7 (38.9%)	6 (40.0%)

^a^*N* = 17. All interview participants were invited to complete the FlymSI-18, even those who did not complete a cognitive interview. Of the 18 participants, one did not complete the FlymSI-18

^b^Self-reported ECOG status: 0 = I have normal activity, and no symptoms; 1 = I have some symptoms, but they do not require that I rest during the day; 2 = My condition requires me to rest for less than 50% of the day; 3 = My condition requires me to rest for more than 50% of the day

^c^Receiving treatment includes active therapy, including trial/study drugs; maintenance therapy; stem cell transplantation; and intravenous immunoglobulin (IVIG)

### Concept elicitation results

**Fig. 3:**
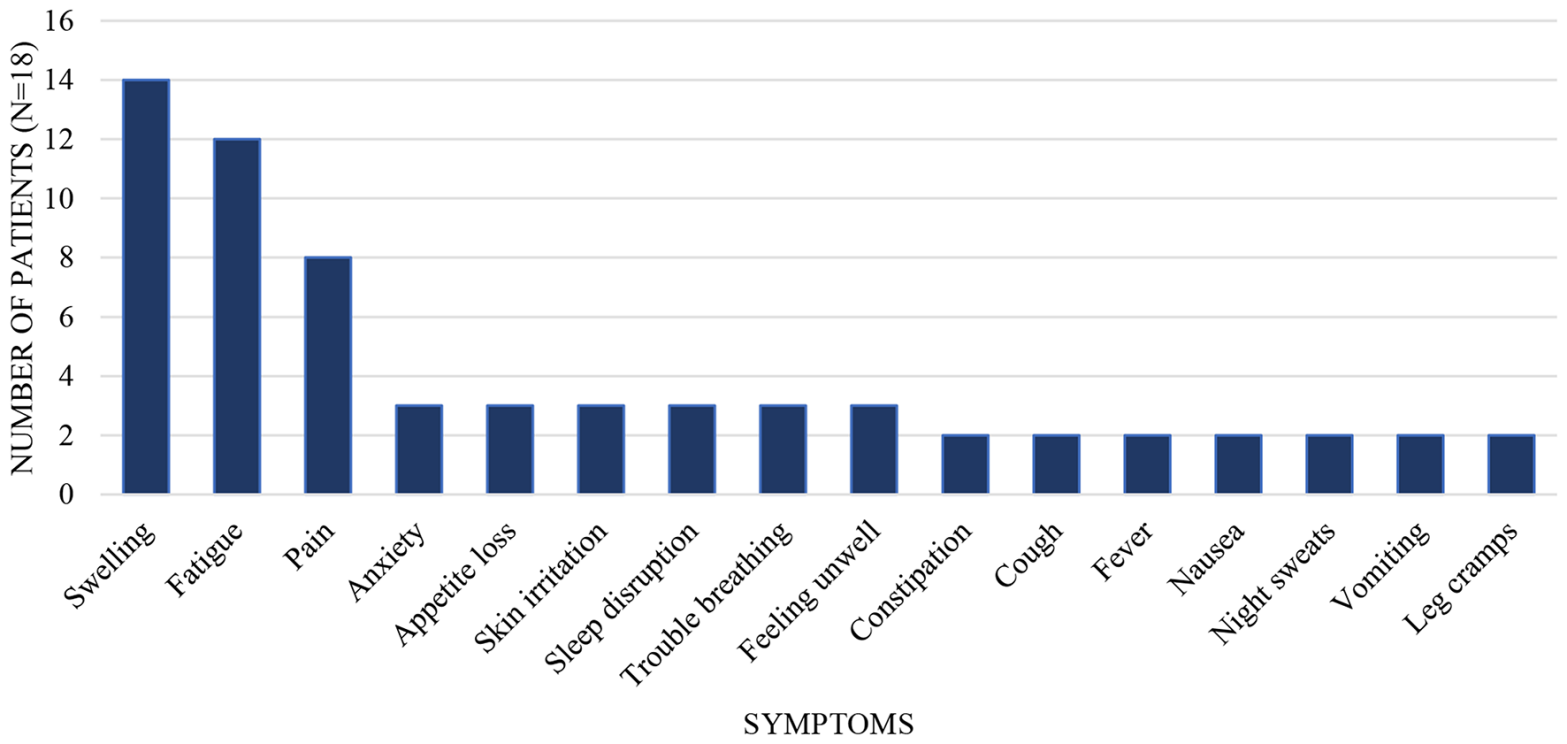
Symptoms reported by two or more iNHL patients (*N* = 18)

Saturation of physical disease related symptoms occurred at interview 18 (Patient 019). When asked to describe their *iNHL symptoms*, patients most often discussed swelling (*n* = 14, 77.8%.), fatigue (*n* = 11, 61.1%), and pain (*n* = 8, 44.4%) (Fig. 3). Twenty additional symptoms were reported; each was endorsed by 1–3 (5.6-16.7%) patients. The following symptoms were mentioned by three patients each: anxiety, appetite loss, rash, sleep disruption, trouble breathing, and malaise. Symptoms reported by two or more patients are shown in Fig. 3. Example quotations for the top three symptoms are found in Table 2. *Treatment side effects* spontaneously mentioned most often were fatigue (*n* = 10, 55.6%), nausea (*n* = 9, 50.0%), hair loss (*n* = 8, 44.4%), cognitive problems (*n* = 6, 33.3%), and weight loss (*n* = 6, 33.3%). *Emotional responses* frequently mentioned included gratitude for life (*n* = 11, 61.1%), worry (*n* = 11, 61.1%), and depressed mood (*n* = 8, 44.4%).

**Table 2:** Patient comments about the most frequently mentioned symptoms

Symptom	Exemplary patient quotes
Swelling	“I was swollen on my left side, but it was subtle. I don’t think anybody noticed but me and the doctors, they could tell, but there was no pain. It didn’t get in the way of any kind of activity. It just looked asymmetrical, swollen.” (015)
	“They did a scan. My liver and my spleen were both enlarged…It looked, my legs looked like I weighed 300 pounds. They were just, the skin was tight. I didn’t hardly have a knee or an ankle, I mean that you could see. It was gross.” (010)
Fatigue	“Then I started to get debilitating fatigue, and it would take me two hours to get dressed. Get out of the shower; I’d have to sit down. Get dressed; I’d have to sit down. Do my makeup; I’d have to sit down…just the fatigue was unbelievable.” (006)
	“It’s a depressing lack of energy. You really want to do something, but all you can do is just kind of just be a lump on the sofa.” (009)
	“[I felt] constantly tired. I mean even when I would sleep, I’d get up and I’d still be tired. It isn’t the type of tired where you want to take a nap, it was just feeling tired. Worn-down, maybe that’s the thing to say, worn down.” (017)
	“This really has not been a disease that’s caused me to change my life much at all…I mean health-wise, outside of feeling a little tired now and then, I’m pretty much okay.” (005)
Pain	“I got a sharp pain in my groin…it wasn’t constant, but it was an intense pain I hadn’t felt before.” (005)
	“I had an abdominal pain, so this swelling of the lymph nodes around the abdomen caused pain…You buckle over. It’s hard to sit straight. It’s hard to stand and walk.” (015)

*Note* Patient identification numbers for each quote are shown in parentheses

### Cognitive interview results

All but three patients who completed a concept elicitation interview also completed a cognitive interview. Of the three who did not complete the cognitive interview, all were unable to due to scheduling conflicts. Demographics for the cognitive interview sample are shown in Table 1. The cognitive interview results revealed good item clarity and respondent understanding. A detailed depiction of the cognitive interview results for all NFlymSI-18 items is shown in Table 3. For every item, at least 14 of the 15 patients (93.3%) said the meaning of the items was clear. Similarly, for every question, nearly all participants found the question easy to answer.

Patients described most DRS-P items as relevant to their iNHL experiences—at least 10 out of 15 (66.7%) patients said the question was relevant to their iNHL experience for all but 2 items. Nine (60.0%) patients reported the item, “I am bothered by lumps or swelling in certain parts of my body (e.g., neck, armpits, or groin)” as relevant. Of the six patients who said the item was less relevant, three did not experience lumps or swelling as a disease symptom, and three explained that they were not bothered by the lumps or swelling experienced. Eight (53.3%) patients reported the item, “I have bone pain” as relevant to their iNHL experiences; those who stated the item was not relevant (*N* = 7, 46.7%) did not have bone pain as a symptom. In summary, two items were seen as less relevant; however, even for these items, over half of patients deemed them relevant.

**Table 3:** Respondent understanding of NFlymSI-18 items (*N* = 15^a^)

Item ID	Item stem	Was the meaning of the question clear?	Do you have any questions about how to answer this question?	Is this question relevant to your experiences with NHL?
**Disease-related symptoms subscale-Physical**				
GP1	I have a lack of energy	No = 0	No = 13	No = 0
		Yes = 15	Yes = 2	Yes = 15
		Missing = 0	Missing = 0	Missing = 0
GP4	I have pain	No = 0	No = 15	No = 2
		Yes = 15	Yes = 0	Yes = 13
		Missing = 0	Missing = 0	Missing = 0
C2	I am losing weight	No = 1	No = 15	No = 3
		Yes = 14	Yes = 0	Yes = 12
		Missing = 0	Missing = 0	Missing = 0
Leu1	I am bothered by lumps or swelling in certain parts of my body (e.g., neck, armpits, or groin)	No = 0	No = 15	No = 6
		Yes = 15	Yes = 0	Yes = 9
		Missing = 0	Missing = 0	Missing = 0
BMT6	I get tired easily	No = 1	No = 14	No = 1
		Yes = 14	Yes = 1	Yes = 14
		Missing = 0	Missing = 0	Missing = 0
BP1	I have bone pain	No = 0	No = 1	No = 7
		Yes = 15	Yes = 14	Yes = 8
		Missing = 0	Missing = 0	Missing = 0
H18	I have trouble concentrating	No = 0	No = 15	No = 3
		Yes = 15	Yes = 0	Yes = 12
		Missing = 0	Missing = 0	Missing = 0
GF5	I am sleeping well	No = 0	No = 15	No = 5
		Yes = 15	Yes = 0	Yes = 10
		Missing = 0	Missing = 0	Missing = 0
C6	I have a good appetite	No = 0	No = 14	No = 2
		Yes = 14	Yes = 0	Yes = 12
		Missing = 1	Missing = 1	Missing = 1
**Disease-related symptoms subscale-Emotional**				
GE6	I worry that my condition will get worse	No = 0	No = 13	No = 0
		Yes = 14	Yes = 1	Yes = 14
		Missing = 1	Missing = 1	Missing = 1
BRM9	I have emotional ups and downs	No = 0	No = 13	No = 2
		Yes = 14	Yes = 1	Yes = 12
		Missing = 1	Missing = 1	Missing = 1
Leu4	Because of my illness, I have difficulty planning for the future	No = 0	No = 12	No = 3
		Yes = 13	Yes = 2	Yes = 10
		Missing = 2	Missing = 1	Missing = 2
Leu5	I feel uncertain about my future health	No = 1	No = 11	No = 3
		Yes = 12	Yes = 2	Yes = 10
		Missing = 2	Missing = 2	Missing = 2
**Treatment side effects subscale**				
GP2	I have nausea	No = 0	No = 13	No = 1
		Yes = 13	Yes = 0	Yes = 12
		Missing = 2	Missing = 2	Missing = 2
N3	I worry about getting infections	No = 0	No = 13	No = 4
		Yes = 13	Yes = 0	Yes = 9
		Missing = 2	Missing = 2	Missing = 2
GP5	I am bothered by side effects of treatment	No = 1	No = 13	No = 0
		Yes = 12	Yes = 0	Yes = 13
		Missing = 2	Missing = 2	Missing = 2
**Function and well-being subscale**				
GF3	I am able to enjoy life	No = 0	No = 13	No = 0
		Yes = 13	Yes = 0	Yes = 13
		Missing = 2	Missing = 2	Missing = 2
GF7	I am content with the quality of my life right now	No = 0	No = 13	No = 0
		Yes = 13	Yes = 0	Yes = 13
		Missing = 2	Missing = 2	Missing = 2

^a^3 of the eligible 18 patients who completed a concept elicitation interview did not complete the cognitive interview

### Mapping to the NFlymSI-18 DRS-P Subscale

Next, the qualitative data from the concept elicitation and cognitive debriefing interviews were mapped to the NFLymSI-18 DRS-P to determine if the subscale covered the most important patient-reported physical symptoms.

#### (GP1) I have a lack of energy

Disease-driven fatigue was mentioned by 61.1% of patients in the concept elicitation interviews, many of whom described fatigue as “lack of energy,” “lethargic,” and “dragging.” Overall, a lack of energy was frequently discussed by patients during concept elicitation and 100% of those who completed a cognitive interview reported this item as relevant to their experience with iNHL.

#### (GP4) I have pain

When reporting symptoms of their iNHL during concept elicitation interviews, 44.4% of patients reported having pain. Most often, patients reported experiencing pain as either resulting from the swollen lymph node itself or resulting from swollen lymph nodes pressing on other organs or nerves. Less common descriptions of pain included one patient who reported pain from leg cramps and another who reported a headache she referred to as a “heavy head.” A majority (86.7%) of those who completed a cognitive interview reported this item as relevant to their experience with iNHL.

#### (C2) I am losing weight

While only one patient reported losing weight as a symptom of iNHL, the literature [14] supports weight loss (a B symptom of iNHL) as a key clinical indicator of active disease. Of those who completed a cognitive interview, 80.0% reported this item as relevant to their experience with iNHL.

#### (Leu1) I am bothered by lumps or swelling in certain parts of my body (e.g., neck, armpits, or groin)

Swelling was the most common symptom reported by patients during the concept elicitation interview; 77.8% of patients identified swelling as a symptom of their iNHL. Importantly, swelling was often the only sign of the disease or of disease progression. There was variability in the significance of swelling for patients in that some described the symptom as being worrisome and others described it as less so, or not at all. Swelling in the form of lumps or bumps (i.e., swollen lymph nodes) often did not cause pain and was, for the most part, not particularly worrisome. Participants like 010 and 007 considered the obvious or visible swelling as most bothersome due to psychological concerns that something may be wrong. On the other hand, participants like 015 reported swelling as less bothersome or noticeable. A few patients reported that swollen lymph nodes caused swelling in other parts of their body. For example, 004 explained that a swollen lymph node in her groin caused her leg and feet to swell and she noted the leg swelling was more bothersome than other swollen lymph nodes. When asked why, she explained, “It was just a visual sign that (something) was not normal.” Of those who completed a cognitive interview, 60.0% reported this item as relevant to their iNHL experience.

#### (BMT6) I get tired easily

As previously noted, fatigue was one of the most reported symptoms of iNHL and was described as debilitating. Many patients revealed sentiments related to getting tired easily, and always feeling tired. On the other hand, for participants like 005, feeling tired was sometimes the only symptom experienced during certain points of the disease trajectory. Of those who completed a cognitive interview, 93.3% reported this item as relevant to their iNHL experience.

#### (BP1) I have bone pain

While pain was one of the top patient-reported symptoms, our sample of patients who completed concept elicitation interviews did not isolate feeling pain in their bones specifically. However, bone pain is clinically relevant as it can be a sign of advanced disease [35, 36] and over half (53.3%) of those who completed a cognitive interview said the item was relevant to their iNHL experience.

#### (H18) I have trouble concentrating

During concept elicitation interviews, no patients spontaneously reported issues with concentration as a symptom of their iNHL. On the other hand, when asked if the item was relevant to their experiences with iNHL, 80.0% of those who completed a cognitive interview said yes.

#### (GF5) I am sleeping well

Three patients reported disrupted sleep when describing their symptoms of iNHL. Patients attributed sleep disruption to symptoms of their iNHL including leg cramps, anxiety, and panic attacks. Of those who completed a cognitive interview, 66.7% reported this item as relevant to their iNHL experience.

#### (C6) I have a good appetite

Three patients reported appetite loss as a symptom of their iNHL. Twelve of the 14 (85.7%) patients who were asked agreed that this item was relevant to their experiences with iNHL.

## Discussion

This study supports the content validity of the NFLymSI-18, including the DRS-P Subscale, for patients with B-cell iNHL. Notably, prior to this study, the content validity of the DRS-P, specifically for B-cell iNHL had not been established. The primary focus of this study is content validity of the DRS-P, which assesses physical symptoms and is most relevant for evaluating disease progression within clinical trials and in clinical practice. Patients in the concept elicitation interview study reported a core set of three physical symptoms: swelling (*n* = 14, 77.8%), fatigue (*n* = 12, 66.7%), and pain (*n* = 8, 44.4%). Additional symptoms were mentioned by smaller numbers of patients. Appetite loss, rash, sleep disruption, trouble breathing, and malaise were each spontaneously mentioned by 3 (16.7%) patients. The diversity of additional symptoms reported by patients is consistent with the heterogeneous symptomology of iNHL.

Mapping of patient-reported symptoms to the NFlymSI-18 DRS-P content showed the instrument includes all commonly expressed physical symptoms of iNHL. For example, the DRS-P includes the most frequently mentioned symptoms of swelling, fatigue, and pain as well as many of the less frequently mentioned symptoms (i.e., appetite loss, sleep disruption). Some less frequently mentioned concerns (i.e., rash, trouble breathing, malaise and fevers) are not included in the NFLymSI-18 DRS-P. However, it is noteworthy that these were concerns raised by a small percentage of patients, supporting the content validity of this subscale. Items representing these symptoms may be used when assessing symptom experiences, as warranted. The cognitive interview results also revealed good item clarity and respondent understanding for the DRS-P. For every item, at least 14 of the 15 participants (93.3%) said the meaning of the items was clear.

This study had several strengths, including a robust approach to content validation where patient experiences are matched to items and the relevance of these items are confirmed with patients living with the condition of interest. However, there are limitations to this study that must be acknowledged. As is common in qualitative research, this study was completed using a relatively small, mostly white, college-educated sample, which, despite evidence of concept saturation, limits generalizability of the results. Interviews were conducted in English, using an English-language version of the NFlymSI instrument. Findings should be confirmed in future non-English language research. Finally, this research was conducted with patients from a single institution who had previously received one or more lines of treatment for follicular lymphoma, marginal zone lymphoma, and lymphoplasmacytic lymphoma/Waldenstrom macroglobulinemia, limiting the generalizability of these results to other iNHL populations.

In summary, we conducted concept elicitation interviews and cognitive interviews with patients with B-cell iNHL. Our findings, and the literature, indicate that although iNHL may occur without symptoms, the presence of symptoms often provides an important signal of disease activity and may adversely affect quality of life. The NFlymSI-18 DRS-P shows excellent content validity for patient-reported symptoms and should be considered a valid tool to understand impacts of iNHL physical symptoms, treatment impacts, and disease progression in many drug development and clinical care settings.

### Electronic supplementary material

Below is the link to the electronic supplementary material.

Supplementary Material 1

Supplementary Material 2

## Data Availability

The datasets generated and/or analyzed during the current study are not publicly available to protect participants’ anonymity.

## Abbreviations

iNHL: Indolent non-Hodgkin’s lymphoma
DRS-P: Disease-Related Symptoms Physical
NFLymSI-18: National Comprehensive Cancer Network-Functional Assessment of Cancer Therapy Lymphoma Symptom Index-18
PRO: Patient-reported outcome
FDA: U.S. Food and Drug Administration
FACIT: Functional Assessment of Chronic Illness Therapy (FACIT) Measurement System
DRS-E: Disease Related Symptoms-Emotional
TSE: Treatment Side Effects
FWB: Functional Well-Being
ECOG: Eastern Cooperative Oncology Group

## References

[1] Ciobanu A, Stanca O, Triantafyllidis I, Lupu A (2013). Indolent lymphoma: diagnosis and prognosis in medical practice. Maedica Sep.

[2] Shankland KR, Armitage JO, Hancock BW (2012) Non-Hodgkin lymphoma. Lancet (London, England) 380(9844):848– 57. 10.1016/s0140-6736(12)60605-9 10.1016/S0140-6736(12)60605-9 22835603

[3] Leukemia & Lymphoma Society. Treatment for indolent NHL subtypes. https://www.lls.org/lymphoma/non-hodgkin-lymphoma/nhl-subtypes/treatment-indolent-nhl-subtypes. Accessed 14 June 2023

[4] Paes FM, Kalkanis DG, Sideras PA, Serafini AN (2010). FDG PET/CT of extranodal involvement in non-hodgkin lymphoma and Hodgkin disease. Radiographics: Rev Publication Radiological Soc North Am Inc Jan.

[5] Pileri SA, Zinzani PL, Went P, Pileri A, Bendandi M (2004). Indolent lymphoma: the pathologist’s viewpoint. Annals Oncology: Official J Eur Soc Med Oncol / ESMO Jan.

[6] Freedman A (2014). Follicular lymphoma: 2014 update on diagnosis and management. Am J Hematol Apr.

[7] McNamara C, Davies J, Dyer M (2012). Guidelines on the investigation and management of follicular lymphoma. Br J Haematol Feb.

[8] Solal-Celigny P, Cahu X, Cartron G (2010). Follicular lymphoma prognostic factors in the modern era: what is clinically meaningful?. Int J Hematol Sep.

[9] National Cancer Institute. Adult Non-Hodgkin lymphoma treatment (PDQ^®^)–Health Professional Version. National Cancer Institute. http://www.cancer.gov/types/lymphoma/hp/adult-nhl-treatment-pdq. Accessed 29 June 2016

[10] Webster K, Cella D (1998) Quality of life in patients with low-grade non-Hodgkin’s lymphoma. Oncology (Williston Park, NY). 12(5):697–714; discussion 714, 717, 721 9597680

[11] Walker MS, Stepanski EJ, Reyes C, Satram-Hoang S, Houts AC, Schwartzberg LS (2011). Symptom Burden and Quality of Life in patients with Follicular Lymphoma undergoing maintenance treatment with Rituximab compared with Observation. Therapeutic Adv Hematol Jun.

[12] MD Anderson Cancer Center. Non-Hodgkin’s lymphoma symptoms, University of Texas MD Anderson Cancer Center. http://www.mdanderson.org/patient-and-cancer-information/cancer-information/cancer-types/non-hodgkins-lymphoma/symptoms/index.html. Accessed 29 June 2016

[13] Leukemia & Lymphoma Society. Non-Hodgkin lymphoma signs and symptoms. Leukemia & Lymphoma Society. https://www.lls.org/lymphoma/non-hodgkin-lymphoma/signs-and-symptoms. 29 Accessed June 2016

[14] American Cancer Society. Signs and symptoms of non-Hodgkin lymphoma. American Cancer Society. http://www.cancer.org/cancer/non-hodgkinlymphoma/detailedguide/non-hodgkin-lymphoma-signs-symptoms. Accessed 27 July 2016

[15] Guidance for industry (2006). : patient-reported outcome measures: use in medical product development to support labeling claims: draft guidance. Health Qual Life Outcomes.

[16] Rosenbloom S, Yount S, Yost K et al (2008) Development and validation of eleven symptom indices to evaluate response to chemotherapy for advanced cancer: measurement compliance with regulatory demands. In: Farquhar ISK, Sorkin A (eds) The value of innovation: impacts on health, life quality, and regulatory research. Emerald Group Publishing Inc, pp 53–66

[17] Cella D, Rosenbloom SK, Beaumont JL (2011). Development and validation of 11 symptom indexes to evaluate response to chemotherapy for advanced cancer. J Natl Compr Cancer Network: JNCCN Mar.

[18] Cella D (1997) FACIT manual: Manual of the Functional Assessment of Chronic Illness Therapy (FACIT) measurement system. Center on Outcomes, Research and Education

[19] Cella DF, Tulsky DS, Gray G (1993). The Functional Assessment of Cancer Therapy scale: development and validation of the general measure. J Clin Oncol Mar.

[20] Webster K, Odom L, Peterman A, Lent L, Cella D (1999) The Functional Assessment of Chronic Illness Therapy (FACIT) measurement system: validation of version 4 of the core questionnaire. Qual Life Res 604–604

[21] Hlubocky FJ, Webster K, Beaumont J (2013). A preliminary study of a health related quality of life assessment of priority symptoms in advanced lymphoma: the National Comprehensive Cancer Network-Functional Assessment of Cancer therapy– lymphoma Symptom Index. Leuk Lymphoma.

[22] Bayer A (2023) Phase III, Randomized, double-blind, controlled multicenter study of intravenous PI3K inhibitor copanlisib in combination with standard immunochemotherapy versus standard immunochemotherapy in patients with relapsed Indolent non-Hodgkin’s Lymphoma (iNHL). https://clinicaltrials.gov/

[23] Matasar MJ, Capra M, Özcan M (2021). Copanlisib plus Rituximab versus placebo plus rituximab in patients with relapsed indolent non-hodgkin lymphoma (CHRONOS-3): a double-blind, randomised, placebo-controlled, phase 3 trial. Lancet Oncol May.

[24] Shaunfield S, Yount SE, Boyken L, Agulnik M, Samant S, Cella D (2021). Optimizing brief, focused assessment of priority symptoms and concerns in recurrent and/or metastatic squamous cell carcinoma of the head and neck: content validation of the Functional Assessment of Cancer Therapy/National Comprehensive Cancer Network Head and Neck Symptom Index-10 (FHNSI-10). Health Sci Rep.

[25] Magasi S, Mallick R, Kaiser K (2013). Importance and relevance of pulmonary symptoms among patients receiving second- and third-line treatment for Advanced non–small-cell Lung Cancer: support for the content validity of the 4-Item Pulmonary Symptom Index. Clin Lung Cancer.

[26] Victorson DE, Anton S, Hamilton A, Yount S, Cella D (2009). A conceptual model of the experience of Dyspnea and Functional limitations in Chronic Obstructive Pulmonary Disease. Value Health: J Int Soc Pharmacoeconomics Outcomes Res.

[27] Eton DT, Shevrin DH, Beaumont J, Victorson D, Cella D (2010). Constructing a conceptual framework of patient-reported outcomes for metastatic hormone-refractory prostate cancer. Value Health Aug.

[28] Willis GB (2005) Cognitive interviewing: a tool for improving questionnaire design. Sage

[29] Bowen GA (2008). Naturalistic inquiry and the saturation concept: a research note. Qualitative Research:QR.

[30] Guest G, Bunce A, Johnson L (2006). How many interviews are Enough? An experiment with data saturation and variability. Field Meth.

[31] Lamoureux RE, Shields A, Stokes J, Yaworsky A, Galipeau N (2015). How many subjects are enough for symptom-focused concept elicitation studies? A retrospective analysis of saturation across twenty-six studies. Value Health.

[32] Glaser BGSA (1967) The discovery of grounded theory: strategies for qualitative research. Aldine

[33] Rothman M, Burke L, Erickson P, Leidy NK, Patrick DL, Petrie CD (2009). Use of existing patient-reported outcome (PRO) Instruments and their modification: the ISPOR Good Research practices for evaluating and documenting content validity for the use of existing instruments and their modification PRO Task Force Report. Value Health.

[34] Aaronson NK, Ahmedzai S, Bergman B (1993). The European Organization for Research and Treatment of Cancer QLQ-C30: a quality-of-life instrument for use in international clinical trials in oncology. J Natl Cancer Inst Mar.

[35] Zheng XQ, Wu YH, Huang JF, Wu AM (2022). Neurophysiological mechanisms of cancer-induced bone pain. J Adv Res Jan.

[36] von Moos R, Costa L, Ripamonti CI, Niepel D, Santini D (2017). Improving quality of life in patients with advanced cancer: targeting metastatic bone pain. Eur J Cancer Jan.

