# Supplementary material for: Content validation of the National Comprehensive Cancer Network/Functional Assessment of Cancer Therapy Lymphoma Symptom Index-18 (NFLymSI-18) in indolent B-cell non-Hodgkin’s lymphoma
Source: J Patient Rep Outcomes. 2024 Jul 9;8:68. doi: 10.1186/s41687-024-00752-6 (PMC11233475; doi:10.1186/s41687-024-00752-6)
Supplement: Supplementary file 1 — Supplementary Material 1 [file 41687_2024_752_MOESM1_ESM.docx]

*Content Validation of the NCCN/FACT-FLymSI-18*

**PATIENT Interview**

**PART i: demographic questions**

1. What is your date of birth? ____/______/_________

Mo Day Year

2. Indicate participant’s gender: ___ Male (1)

___ Female (2)

3. Do you consider yourself to be of Hispanic or Latino Origin? ___ No (0)

___ Yes (1)

4. What race do you consider yourself to be? (check all that apply):

___ White (1)

___ African American or Black (2)

___ Asian (3)

___ American Indian or Alaska Native (4)

___ Native Hawaiian or other Pacific Islander (5)

___ Other (please specify_______________) (6)

5. What is the highest year of education you have completed (check one)?

___ 8th grade or less (1)

___ Some high school (2)

___ High school grad/GED (3)

___ Some college/Technical degree/AA (4)

___ College degree (BA/BS) (5)

___ Advanced degree (MA, PHD, MD) (6)

**PART II. HQOL CONCERNS**

**(TURN ON RECORDER)** Thank you for agreeing to share your experiences. We are interested in knowing about the symptoms, side effects, and any concerns you have that relate to your experience with non-Hodgkin’s lymphoma (NHL). We refer to the sum of all of your symptoms, side effects and other concerns as your health-related quality of life.

1. Please consider anything and everything that relates to your quality of life as you live with NHL. What do **you** think is important in terms of your quality of life? *(Interviewer—probe as needed to ensure that the patient provides a clear description of each concern.)*

*Interviewer lists all concerns provided by the patient:* Ranking:

____________________________________________________ ________

____________________________________________________ ________

____________________________________________________ ________

____________________________________________________ ________

____________________________________________________ ________

____________________________________________________ ________

____________________________________________________ ________

____________________________________________________ ________

**____________________________________________________ ________**

____________________________________________________ ________

____________________________________________________ ________

____________________________________________________ ________

____________________________________________________ ________

____________________________________________________ ________

____________________________________________________ ________

1. Next, I would like you to think about the importance of each of these concerns.

On a 0-10 scale, with 0 = ***Not at all important*** and 10 = ***Extremely important***, please rate the importance of each of these concerns to your health-related quality of life. We’ll work through each of the concerns, one-by-one.

*(Read each of the concerns listed under Question 1 and ask patient to rank it from 0-10.Indicate their ranking next to the items above*.)

For the rest of the interview we’re going to be talking about some of these concerns in more detail. We’ll break our discussion up into the physical symptoms of your disease, the emotional impact of NHL, and the treatment side effects that you’ve experienced.

3. We’ll start by focusing on the physical symptoms of NHL that you’ve experienced. What physical symptoms of NHL have you experienced? (*Interviewer—probe as needed to ensure that the patient provides a clear description of each symptom.)*

*List all physical symptoms provided by the patient:* Ranking:

____________________________________________________ ________

____________________________________________________ ________

____________________________________________________ ________

____________________________________________________ ________

____________________________________________________ ________

____________________________________________________ ________

____________________________________________________ ________

____________________________________________________ ________

**____________________________________________________ ________**

____________________________________________________ ________

____________________________________________________ ________

____________________________________________________ ________

4. Next, I would like you to think about the importance of each of these physical symptoms. On a 0-10 scale, with 0 = ***Not at all important*** and 10 = ***Extremely important***, please rate the importance of each of these physical symptoms to your health-related quality of life. We’ll work through each of the concerns, one-by-one.

5. Next, I’d like your input on the emotional impact of NHL. Could you tell me how NHL has affected you emotionally? (*Interviewer—probe as needed to ensure that the patient provides a clear description of each emotional impact.)*

*List all emotional impacts provided by the patient:* Ranking:

____________________________________________________ ________

____________________________________________________ ________

____________________________________________________ ________

____________________________________________________ ________

____________________________________________________ ________

____________________________________________________ ________

____________________________________________________ ________

____________________________________________________ ________

**____________________________________________________ ________**

____________________________________________________ ________

____________________________________________________ ________

____________________________________________________ ________

6. Now please rate each of these emotional concerns using the same 0-10 scale, with 0 = ***Not at all important*** and 10 = ***Extremely important***.

7. Finally, we’d like to hear your thoughts on the treatment side effects you’ve experienced. Could you tell me about the side effects you’ve experienced from treatment for your NHL? (*Interviewer—probe as needed to ensure that the patient provides a clear description of each side effect.)*

*List all side effects provided by the patient:*  Ranking:

____________________________________________________ ________

____________________________________________________ ________

____________________________________________________ ________

____________________________________________________ ________

____________________________________________________ ________

____________________________________________________ ________

____________________________________________________ ________

____________________________________________________ ________

**____________________________________________________ ________**

____________________________________________________ ________

____________________________________________________ ________

____________________________________________________ ________

8. Now please rate each of these emotional concerns using the same 0-10 scale, with 0 = ***Not at all important*** and 10 = ***Extremely important***.

9. Is there anything else you’d like to add about the effect of NHL on your quality of life?

**PART III: REVIEW OF THE NCCN-FACT FLymSI-18**

(Have patient complete the NCCN-FACT FLymSI-18.)

You just completed a questionnaire called the National Comprehensive Cancer Network-Functional Assessment of Cancer Therapy Lymphoma Symptom Index-18 (NCCN-FACT FLymSI-18). We would appreciate your feedback regarding this questionnaire in order to determine which questions are the most appropriate to be asking patients lymphoma.

First, I’d like to ask you some general questions about the questionnaire.

1. What time frame did you consider when answering these questions?

2. Were the instructions clear to you? ___ No (0)

___ Yes (1)

If no🡪 What was unclear about the instruction?

3. These questions ask you to rank your response from Not At All to Very Much.

3a. Did those response options make sense to you? ___ No (0)

___ Yes (1)

If no🡪 why not?

3b. Was it easy to respond using those options? ___ No (0)

___ Yes (1)

If no🡪 why not?

3c. How did you distinguish “a little bit,” “somewhat,” and “quite a bit” from each other? (Probe, if participant struggles to answer: Could you use one question as an example and explain how you distinguished your response from the other response options?)

3d. Did this format provide enough response choices? ___ No (0)

___ Yes (1)

3e. Did this format provide too many response choices? ___ No (0)

___ Yes (1)

4. Would you say the length of the questionnaire was: ___ Too short (1)

___ Too long (2)

___ Just about right (3)

Next, I’m going to ask you a series of questions about each of the items on the questionnaire.

| **Item** | **What kinds of things did you think about when you answered the question? (i.e. How did you come to the answer you gave)?** | **How would you state the question in your own words? (If patient struggles, remind them that it’s ok if they would say it the same way as it’s written.)** | **What does (term[s]) mean to you?** | **Was the meaning of the question clear to you?**  **Yes/NO**  **If no, what was unclear?** | **Do you have any questions about how to answer this question?**  **Yes/NO**  **If yes, what questions did you have?** | **How confident are you that you can provide an accurate answer to this question?**  **1) Very Confident**  **2) Confident**  **3) Not at all confident** |
| --- | --- | --- | --- | --- | --- | --- |
| I have a lack of energy. |  |  | *Lack of Energy* |  |  |  |
| I have pain. |  |  | *Pain* |  |  |  |
| I am losing weight. |  |  | *Losing weight* |  |  |  |
| I am bothered by lumps or swelling in certain parts of my body (e.g., neck, armpits, or groin) |  |  | *Lumps or swelling in certain parts of my body* |  |  |  |
| I get tired easily. |  |  | *Get tired easily* |  |  |  |
| I have bone pain. |  |  | *Bone pain* |  |  |  |
| I have trouble concentrating. |  |  | *Trouble concentrating* |  |  |  |
| I am sleeping well. |  |  | *Sleeping well* |  |  |  |
| I have a good appetite. |  |  | *A good appetite* |  |  |  |
| I worry that my condition will get worse. |  |  | *Worry that my condition will get worse* |  |  |  |
| I have emotional ups and downs. |  |  | *Emotional ups and downs* |  |  |  |
| Because of my illness, I have difficulty planning for the future. |  |  | *Difficulty planning for the future* |  |  |  |
| I feel uncertain about my future health. |  |  | *Uncertaint about my future health* |  |  |  |
| I have nausea. |  |  | *Nausea* |  |  |  |
| I worry about getting infections. |  |  | *Infections* |  |  |  |
| I am bothered by side effects of treatment. |  |  | *Side effects of treatment* |  |  |  |
| I am able to enjoy life. |  |  | *Enjoy life* |  |  |  |
| I am content with the quality of my life right now. |  |  | *Quality of life* |  |  |  |

**Summary Questions:**

1. In general, what do you think the questions you answered are getting at? *(Interviewer may prompt patient with “testing” or “meaning” if needed.)*
2. Please take a moment to look over the questions again. Do these questions, in your opinion, capture your experience?

___ No (0)

___ Yes (1)

If No🡪 Why not?

____________________________________________________

____________________________________________________

____________________________________________________

____________________________________________________

1. Are there any other important questions we didn’t ask you?

4. Is there anything else that you would like to suggest that would help us to improve these questions for future use?
