# Supplementary material for: Content validation of the National Comprehensive Cancer Network/Functional Assessment of Cancer Therapy Lymphoma Symptom Index-18 (NFLymSI-18) in indolent B-cell non-Hodgkin’s lymphoma
Source: J Patient Rep Outcomes. 2024 Jul 9;8:68. doi: 10.1186/s41687-024-00752-6 (PMC11233475; doi:10.1186/s41687-024-00752-6)
Supplement: Supplementary file 2 — Supplementary Material 2 [file 41687_2024_752_MOESM2_ESM.docx]

| **Consolidated criteria for reporting qualitative studies (COREQ): 32-item checklist** | | |
| --- | --- | --- |
| No. | Item | Response |
| **Domain 1: Research team and reflexivity** | | |
| *Personal characteristics* | | |
| 1. | Interviewers | KK, SS, and LB conducted all the interviews |
| 2. | Credentials | KK has a PhD; SS has a PhD; LB has a bachelor’s degree |
| 3. | Occupation | KK and SS are HRQoL researchers and Research Associate Professors in a medical school. At the time of the study, LB was a Study Coordinator with experience in qualitative data analysis. |
| 4. | Gender | All interviewers are female. |
| 5. | Experience and training | KK and SS are highly experienced qualitative researchers. LB was trained to conduct interviews on this project and was closely supervised, including shadowing, reverse shadowing, and regular debriefing following interviews with KK and/or SS. |
| *Relationship with participants* | | |
| 6. | Relationship established | The interviewers had no prior relationships with study participants, but established rapport during the recruitment and consent process. |
| 7. | Participant knowledge of interviewer | Participants did not know the interviewer prior to the day of recruitment. |
| 8. | Interviewer characteristics | (Described in Items 2, 3, 4, and 5) |
| **Domain 2** | | |
| *Theoretical framework* | | |
| 9. | Methodological orientation and theory | Methods based on guidelines established by FDA and others for eliciting patient feedback to evaluate questionnaires for clinical use; constant comparative approach to analysis |
| *Participant selection* | | |
| 10. | Sampling | Purposive sampling method |
| 11. | Method of approach | A clinical recruitment specialist reviewed medical records at a large academic cancer center in the Midwest US to identify patients with a confirmed iNHL diagnosis who had received one or more lines of treatment. Eligible patients were approached in clinic prior to or following a scheduled appointment by a study team member, who explained the study using an IRB-approved recruitment script and obtained written informed consent from interested patients. |
| 12. | Sample size | A total of 18 participants were in this study |
| 13. | Non-participation | 22 individuals were approached to enroll in the study. Four individuals declined due to disinterest in participating. |
| *Setting* | | |
| 14. | Setting of data collection | Interviews were held in the patient’s infusion room while receiving treatment or in private meeting rooms. |
| 15. | Presence of non-participants | Some interviews were conducted while the participant as undergoing treatment in an infusion room. Therefore, interviews were briefly interrupted by clinical staff for routine clinical purposes for 5 participants. One participant had his wife present who served to confirm details (e.g., dates and timelines) but did not contribute to the list of symptoms/concerns reported by the participant. |
| 16. | Description of sample | Mean age of the 18 patients who completed concept elicitation interviews was 66.9 years. Eighty-three percent of the sample was White. Fifty-six percent of the sample was male. Most participants (66.7%) had a college-level or advanced degree. Eighty-nine percent of the sample had a self-reported Eastern Cooperative Oncology Group (ECOG) Performance Status ≤1. |
| *Data collection* | | |
| 17. | Interview guide | The interview guide was modeled after prior research with similar aims. |
| 18. | Repeat interviews | The cognitive debriefing interview occurred after the concept elicitation interview. Neither of these interviews were repeated for any individual participant. |
| 19. | Audio/visual recording | Interviews were audio-recorded, transcribed verbatim, and de-identified prior to analysis. |
| 20. | Field notes | Interviewers took detailed field notes during the interview. After the interview, field notes entered into an Excel file for each study participant. |
| 21. | Duration | The interviews lasted approximately 45 minutes. |
| 22. | Data saturation | Saturation was assessed by evaluating the point at which no new relevant themes emerged for three consecutive interviews. Saturation occurred at interview 18 (Patient 019). |
| 23. | Transcripts returned | Transcripts were not returned to participants. |
| **Domain 3: Analysis and findings** | | |
| *Data analysis* | | |
| 24. | Number of data coders | Three data analysts independently reviewed and coded three transcripts in NVivo10 using the draft codebook, noting missing or problematic codes. In group meetings, the coded transcripts were collectively reviewed, discrepancies were discussed, and the codebook was refined. After finalizing the codebook, two team members independently coded the remaining transcripts. The coding review process involved two team members individually reviewing each code. |
| 25. | Description of the coding tree | First, detailed field notes were reviewed and lists of quality-of-life concerns, symptoms, side effects, and emotional issues were compiled, redundant concepts were removed, resulting in a preliminary codebook. The codebook was refined iteratively during analysis via team discussion. |
| 26. | Derivation of themes | Themes were derived inductively from the data. |
| 27. | Software | NVivo 10 and Microsoft Excel |
| 28. | Participant checking | Participants did not provide feedback on the research results. |
| *Reporting* | | |
| 29. | Quotations presented | Quotations are included in the manuscript. |
| 30. | Data and findings consistent | There is consistency between the data and the research findings. |
| 31. | Clarity of major themes | Major and minor themes are clearly delineated in the Results and Discussion section of the manuscript. |
